# A Cross-Sectional Observation on Maximal Eccentric Hamstring Strength in 7- to 15-Year-Old Competitive Alpine Skiers

**DOI:** 10.3390/biology10111128

**Published:** 2021-11-03

**Authors:** Kirsten Kiers, Lynn Ellenberger, Marie Javet, Björn Bruhin, Walter O. Frey, Jörg Spörri

**Affiliations:** 1 Sports Medical Research Group, Department of Orthopaedics, Balgrist University Hospital, University of Zurich, 8008 Zurich, Switzerland; kirsten.kiers@gmail.com (K.K.); le@hest.ethz.ch (L.E.); walterofrey@hin.ch (W.O.F.); 2 University Centre for Prevention and Sports Medicine, Department of Orthopaedics, Balgrist University Hospital, University of Zurich, 8008 Zurich, Switzerland; 3 Section for Elite Sport, Swiss Federal Institute of Sport Magglingen, 2532 Magglingen, Switzerland; marie.javet@baspo.admin.ch; 4 Swiss-Ski, 3074 Muri bei Bern, Switzerland; bjoern.bruhin@swiss-ski.ch

**Keywords:** skiing, athletes, biological maturity status, conditioning, injury prevention, neuromuscular performance, physical fitness, testing

## Abstract

**Simple Summary:**

Competitive alpine skiing is a sport with frequent occurrence of severe knee injuries, and it is well known that the hamstring muscles play an important role in preventing these injuries. The aim of this study was to assess the maximal strength capacity for braking, the downward movement during Nordic Hamstring Exercises, the so called maximal eccentric hamstring strength, in 7- to 15-year-old skiers. Absolute strength values were greater in skiers under 15 years old (U15) skiers than in those under 10 years old (U10), as well as in U15 males compared to their female counterparts. There were no sex differences in U10 skiers. Absolute strength values were generally dependent on age and biological developmental stage, but this dependence was considerably attenuated when body weight was considered. This should be kept in mind when testing athletes around the growth spurt.

**Abstract:**

Severe knee injuries are common in alpine skiing and the hamstring muscles are known to counteract the anterior tibial displacement that typically accompanies major injury mechanisms. This study aimed to assess the Maximal Eccentric Hamstring Strength (MEHS) of youth competitive alpine skiers during Nordic Hamstring Exercise (NHE) in terms of dependence of sex, age and biological maturation. A total of 246 7- to 15-year-old skiers were tested with respect to their MEHS using an NHE-based measurement device (Vald Performance, Newstead, Australia). Significantly greater absolute MEHS was observed in skiers of the under 15 years (U15) category compared to skiers under 10 years old (U10) (227.9 ± 61.1 N vs. 142.6 ± 28.9 N; *p* < 0.001), also when grouped by sex. Absolute MEHS was revealed to be lower in U15 females compared to males (213.5 ± 49.0 N vs. 241.9 ± 68.4 N; *p* = 0.001); in U10 skiers there was no sex difference. For all age groups and sexes, absolute MEHS values were significantly correlated with age and biological maturation (*p* < 0.001). However, when normalized to body weight such associations disappeared, which is why this is strongly recommended when testing around their growth spurt. Overall, this study established sport-specific normative reference data that may be of interest to researchers and sport practitioners alike.

## 1. Introduction

Competitive alpine skiing is a sport with a relatively high injury risk compared to other sports, which also at youth level [1,2,3]. Knee injuries are the most common injuries in alpine skiers in general, with anterior cruciate ligament (ACL) ruptures being the most frequent injuries in competitive alpine youth skiers [1,2,3]. Thus, the prevention of ACL injuries should be an integral part of the training of skiers, starting already in the early stages of their career.

Not only in competitive alpine skiing, but also in other sports, is there a growing interest in youth injury prevention, as it has been shown that specially tailored prevention programs, such as FIFA 11+, can be highly effective [4,5,6,7,8]. In addition, increased knowledge about the skiing-related mechanisms of ACL injury has been gained in the last decade, with three typical mechanisms described: the ‘slip-catch’, ‘landing back-weighted’ and ‘dynamic snowplow’ mechanisms [9,10,11,12]. These mechanisms mainly occur during turns and jump landings [10,13].

In all three mechanisms, but especially in the landing back-weighted, the hamstring muscles play an important role in terms of a modifiable risk factor [12]. Hamstring muscle strength is assumed to prevent knee injuries since forces in the ACL are significantly reduced when increasing the hamstring load [14,15,16]. When the skier becomes out of balance during a jump and lands backwards on their ski tails, tibiofemoral compression and a boot- and/or quadriceps-induced anterior drawer of the tibia relative to the femur occur [10]. Thus, a rapid and maximally eccentric contraction of the hamstring muscles, may counteract such anterior drawer and, therefore, may prevent the skier from an ACL tear [9,12], and specific training including eccentric exercises to strengthen the hamstrings, such as Nordic hamstring exercises, may be a promising approach. Indeed, such a hypothesis is supported by emerging evidence that neuromuscular injury prevention programs (and Nordic Hamstrings Exercises in particular) have been effective in reducing the risk of ACL injuries in sports other than alpine ski racing [17].

To determine injury-relevant hamstring strength capacities in youth competitive alpine skiers, a recent study concluded maximal bilateral eccentric hamstring tests were more suitable than unilateral isometric hamstring tests [18]. Such a maximal bilateral eccentric hamstring test can easily be implemented with a Nordic Hamstring Exercise (NHE)-based test device, such as the NordBord (Vald Performance, Newstead, Australia). This approach has already been followed in a recent study with competitive alpine skiers [19]. However, this study primarily focused on skiers of the under 16 years (U16) category up to the elite level, while, to date, there is no study providing maximal eccentric hamstrings strength (MEHS) reference values for younger skiers (e.g., aged 7 to 15 years).

Therefore, the aims of the current study were three-fold: (1) to screen and compare two groups of youth competitive alpine skiers (U15 and U10 athletes) with respect to Maximal Eccentric Hamstring Strength (MEHS) during NHEs; (2) to assess potential sex differences; (3) to investigate potential relationships of MEHS with age, anthropometrics and biological maturation.

## 2. Materials and Methods

### 2.1. Study Design, Setting and Participants

The present cross-sectional study is based on an anonymized data set of 246 7- to 15-year-old competitive alpine skiers (121 females and 125 males) originally collected as part of a physical fitness competition at the final event of the ‘Swiss-Ski Smile Pass Challenge’ during the off-season period in the summer of 2020 in Switzerland. Inclusion criterion was participation in the aforementioned event, and none of the participants were excluded based on any other criteria. The reuse of this anonymized dataset was approved by the Cantonal Ethics Committee KEK Zurich (KEK-ZH-NR: 2021-01044), and was judged not to fall under the scope of the Human Research Act (HRA), which meant that no written informed consent was required from the participants.

### 2.2. Data Collection and Evaluation

#### 2.2.1. Baseline Data and Determination of Biological Maturity

There was a guided 10’ warm-up at the beginning of the event. During the event, all skiers underwent accompanying baseline assessments including the determination of chronological age, body weight (1 kg, Seca, Hamburg, Germany), body height and sitting height (1 cm, determined by measuring tape). Maturity offset was obtained using the non-invasive, anthropometric methodology proposed by Mirwald et al. [20], which predicts the age at peak height velocity (APHV) and had been validated in previous studies [21,22,23]. To calculate maturity offset, sex-specific formulas were used that build on the following input data: chronological age, body weight, body height, sitting height and leg length (body height—sitting height). Maturity offset thereby represents a point in time before or after the age at peak height velocity (APHV). Maturity offset was calculated for the group of U15 skiers and not U10 skiers, assuming that the Mirwald equation has limited validity for substantial departures from APHV [24,25]. Average APHV for female skiers was previously reported as 12.44 ± 0.45 years, and 14.36 ± 0.66 years for male skiers [2].

#### 2.2.2. Maximal Eccentric Hamstrings Strength (MEHS)

Absolute MEHS was obtained using the NHE-based NordBord test device (Vald Performance, Newstead, Australia). NHEs are typical training content of youth competitive alpine skiers recommend by the national skiing association Swiss-Ski, and participants were additionally familiarized with this type of exercise by social media challenges before the event. Reliability and application of the NordBord test device within different athlete populations has already been investigated and reported by various studies [26,27,28]. According to Opar et al. [27] the NordBord device possesses high to moderate reliability (intraclass correlation coefficient = 0.83–0.90; typical error, 21.7–27.5 N; typical error as a coefficient of variation, 5.8–8.5%; minimal detectable change at a 95% confidence level, 60.1–76.2 N).

The test task was as follows: athletes were positioned with their knees on a padded board, without shoes, with the ankles secured by braces just above the lateral malleoli. Athletes were instructed to keep their shoulders, hips and knees in one line with the arms crossed in front of their body while gradually moving forward as slow as possible and resisting the movement with both legs. They had to move forward until they could no longer hold the position. They then used their hands and arms to brace their fall and returned to the starting position. Correct exercise execution was controlled by the test instructor, who gave immediate feedback and repeated the test in case of deviations from the specified quality criteria or movement speed. All athletes performed three trials and the best left and right maximum values of the three repetitions were used for further data analysis. MEHS was measured with the ankle braces. Relative MEHS was obtained by normalizing the absolute maximal MEHS (N) by body weight (kg).

### 2.3. Statistical Analysis

IBM SPSS software (Version 26) was used for statistical analysis. Normal distribution of the data was checked using the Shapiro–Wilk test, graphical techniques (i.e., histograms and quantile–quantile plots) and shape parameters (i.e., skewness and kurtosis coefficients). In case of normal distribution, data were analyzed with standard parametric tests. Skewness and kurtosis values below common reference boundaries of a clear deviation from normality (skewness > 2 and kurtosis > 7 according to West et al. [29]) were considered acceptable. All accompanying baseline and biological maturity data were evaluated with respect to the potential age group (U10 vs. U15), as well as sex (female vs. male) differences by the use of a multivariate analysis of variance (MANOVA) at *p* < 0.05, with Bonferroni correction for pairwise comparisons. Age group (U10 vs. U15), and sex (female vs. male) differences in absolute MEHS values were analyzed by a two-way analysis of variance (ANOVA) at *p* < 0.05; again, with Bonferroni correction. Pairwise comparisons were additionally illustrated by mean and 95%CI plots. Correlations of absolute and relative (i.e., body weight normalized) MEHS, with age and biological maturation were tested using the Pearson’s correlation coefficient (r) and the coefficient of determination (R^2^). The level of significance was set at *p* < 0.05.

## 3. Results

### 3.1. Baseline Data and Biological Maturation

On a multivariate level, significant differences between U10 and U15 skiers (*p* < 0.001; partial eta^2^ = 0.759), and between female and male skiers (*p* < 0.001; partial eta^2^ = 0.955) were found. There was an interaction effect for age group * sex group (*p* < 0.001; partial eta^2^ = 0.319). On a univariate level, age group comparisons were significant for age (*p* < 0.001; partial eta^2^ = 0.745), height (*p* < 0.001; partial eta^2^ = 0.631), weight (*p* < 0.001; partial eta^2^ = 0.585), BMI (*p* < 0.001; partial eta^2^ = 0.362), maturity offset (*p* < 0.001; partial eta^2^ = 0.715), and APHV (*p* < 0.001; partial eta^2^ = 0.415). Univariate analyses for sex comparisons revealed significant results for height (*p* = 0.018; partial eta^2^ = 0.023), maturity offset (*p* < 0.001; partial eta^2^ = 0.303), and APHV (*p* < 0.001; partial eta^2^ = 0.725), but not for age (*p* = 0.347; partial eta^2^ = 0.004), weight (*p* = 0.356; partial eta^2^ = 0.004) and BMI (*p* = 0.396; partial eta^2^ = 0.003). Detailed descriptive statistics for all age and sex groups can be found in Table 1.

### 3.2. Differences in Absolute MEHS with Respect to Age and Sex

**Figure 1:**
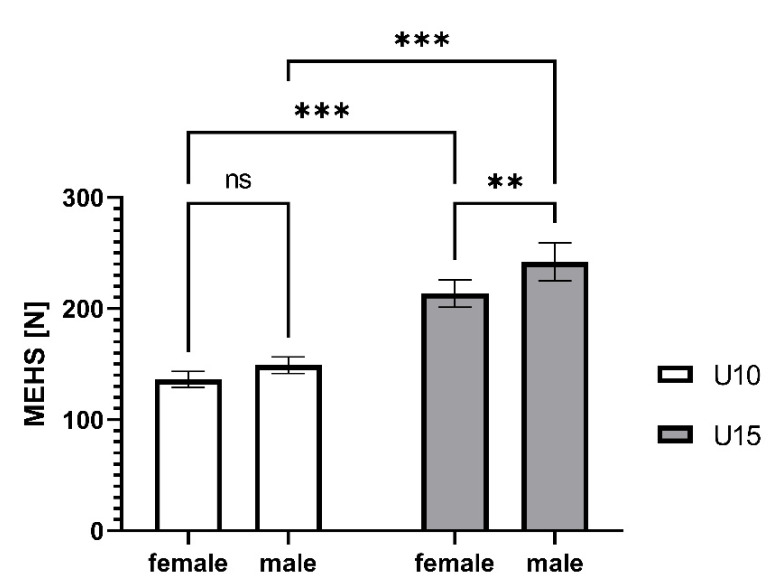
Maximal eccentric hamstring strength (MEHS) separated by age group and sex. Data are expressed as the mean and 95%CI. Level of significance based on a two-way ANOVA: ns not significant; ** *p* < 0.01; *** *p* < 0.001.

Univariate ANOVA revealed significant differences in absolute MEHS between U10 and U15 skiers (*p* < 0.001; partial eta^2^ = 0.454), and between female and male skiers (*p* = 0.001; partial eta^2^ = 0.047). However, there was no interaction effect for age group * sex (*p* = 0.199; partial eta^2^ = 0.007). Detailed pairwise comparisons are highlighted in Figure 1.

### 3.3. Association between MEHS, Sex, Age and Maturity Offset

**Figure 2:**
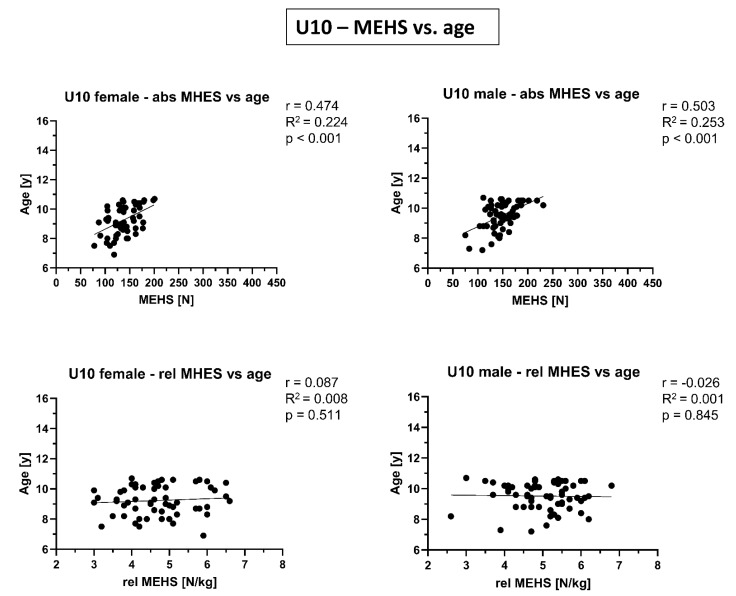
Correlations of absolute (abs) and relative (rel) maximal eccentric hamstring strength (MEHS) with age for competitive alpine skiers under 10 years old (U10) separated by sex.

**Figure 3:**
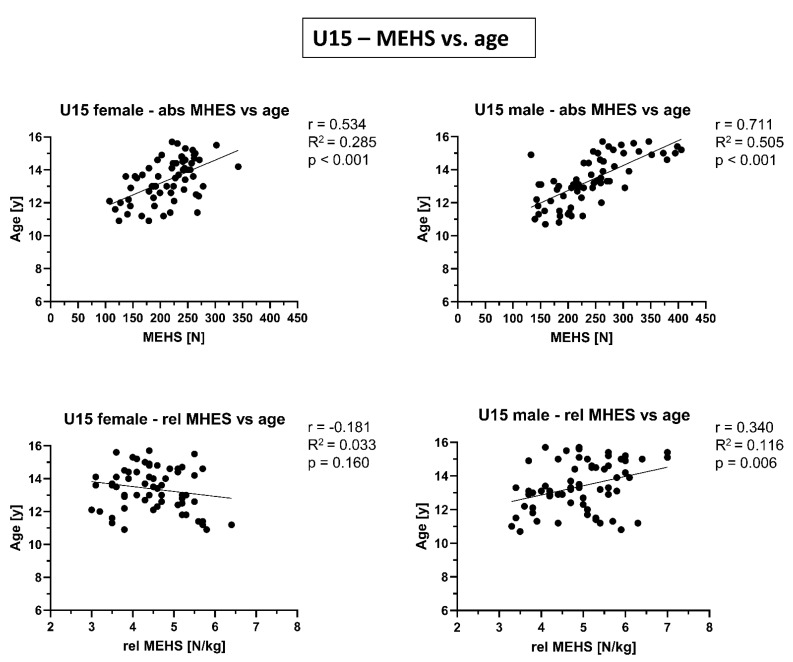
Correlations of absolute (abs) and relative (rel) maximal eccentric hamstring strength (MEHS) with age for competitive alpine skiers under 15 years old (U15) separated by sex.

**Figure 4:**
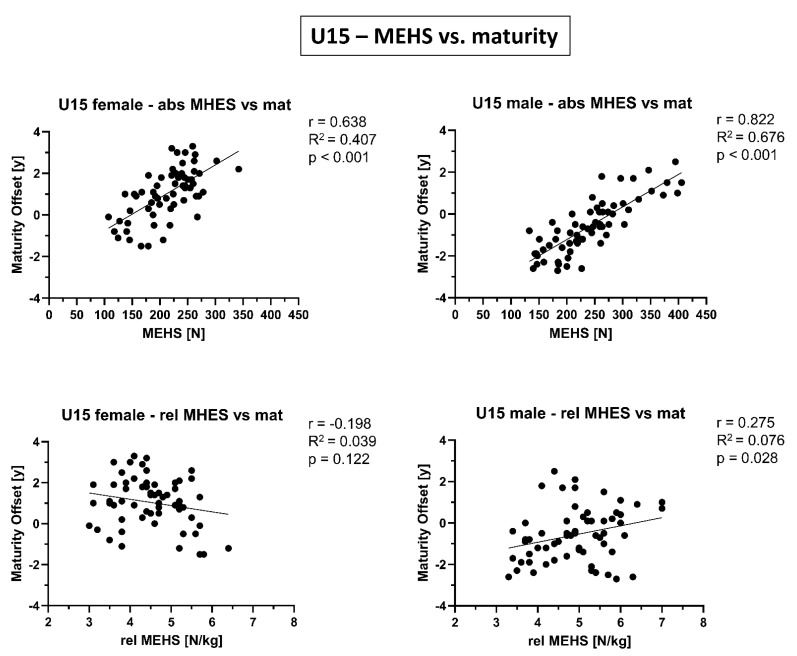
Correlations of absolute (abs) and relative (rel) maximal eccentric hamstring strength (MEHS) with maturity offset for competitive alpine skiers under 15 years old (U15) separated by sex.

In Figure 2, Figure 3 and Figure 4, correlations between absolute/relative MEHS and age/biological maturity are presented. While for the absolute MEHS values of both age groups and sexes were significantly correlated with age and biological maturation (*p* < 0.001), such relations were not present (or at least strongly weakened) for relative, i.e., body weight normalized, MEHS values.

## 4. Discussion

The main findings of the study were: (1) absolute MEHS values during NHE were greater in the U15 skiers than in U10 skiers, also when grouped by sex; (2) absolute MEHS was revealed to be lower in U15 females compared to males; in U10 skiers there was no sex difference; and (3) absolute MEHS values of both age groups and sexes were significantly correlated with age and biological maturation, but were not present (or at least considerably weakened) when normalizing MEHS by body weight.

### 4.1. Absolute MEHS in U10 vs. U15 Youth Skiers

This study provided reference data for MEHS in female and male skiers aged 7 to 15 years and revealed a difference between U15 and U10 skiers. A similar study was carried out by Franchi et al. [19], which, however, focused on skiers from the youth up to the elite level. While the MEHS reference data for the overlapping portions of the two independent study samples (i.e., U15 skiers) in this and the study by Franchi et al. [19] are of similar magnitude, data on younger athletes have so far been lacking. To date, no previous studies on MEHS values (measured during NHE) for U10 athletes in sports other than skiing are available. In adult athletes, however, greater MEHS values were observed in alpine skiers compared to soccer, rugby and Australian football athletes [19,26,28,30]. As stated by Franchi et al. [19], this is probably due to the high force production of the knee extensors, the antagonists of the hamstring muscles, which is typical for alpine skiing. Accordingly, the hamstrings muscles are also specifically trained in order to reduce adverse muscular imbalances.

### 4.2. Absolute MEHS in Female vs. Male Youth Skiers

Absolute MEHS was revealed to be lower in U15 females compared to males; in U10 skiers there was no sex difference. This may suggest that when youth skiers are maturing, i.e., moving closer to APHV, sex differences start manifesting. In the U15 skiers, the mean maturity offset was past APHV with 0.2 ± 1.5 y. In general, females reached maturity offset before males. In this study, the average maturity offset in females was already past APHV (1.0 ± 1.2 y) and for males it was before APHV (−0.6 ± 1.3 y). Therefore, the difference between male and female U15 skiers is probably due to the sex-dependent developmental differences that occur around the growth spurt. Maturity offset was not calculated for the younger, U10 skiers, as it is known that its validity decreases with increasing distance from the APHV.

It is entirely reasonable that sex differences manifest during puberty and that athletes should therefore train and compete separately after a certain development point. This is based primarily on the consideration that sex hormones affect boys and girls differently during puberty, as well as the significant physical difference reported by Philippaerts et al. [31] according to which muscle mass and strength increase more in boys than in girls. In addition to this difference in muscle mass, neuromuscular control also differs between males and females, with greater neuromuscular control in males [32,33]. It is therefore plausible to assume that a difference in absolute MEHS between males and females is not yet noticeable in the U10 skiers. Thus, when testing and interpreting the physical fitness of young athletes around the growth spurt, it is important to consider sex differences in muscle mass, APHV and neuromuscular control.

### 4.3. Relationship between Age, Biological Maturation and MEHS

For the absolute MEHS values of all age groups and both sexes, there were significant associations with age and biological maturation. This suggests that individual differences may be due to age and maturity and not only to training factors, with hamstring strength increasing with age and maturity. A stronger correlation between absolute MEHS and age was found in U15 male skiers than U15 female skiers and younger (U10) skiers. This correlation in male U15 skiers had an R^2^ value of 0.505, explaining 50.5% of the variability of MEHS by age. Overall, this suggests a stronger influence of age in males and older skiers.

As described above, the increase in muscle mass in males is one of the most important sex-specific changes during puberty and may explain the stronger correlation between hamstrings strength and age in males compared to females [31]. In contrast, Franchi et al. [19] described that the correlation between age and MEHS was not found in the elite athletes (17–28 years old). Thus, the influence of age on MEHS seems to increase around the growth spurt, but to decrease or stabilize thereafter.

A strong correlation between absolute MEHS and maturity offset was found in female U15 skiers and an even stronger one in male U15 skiers. These correlations showed R^2^ values of up to 0.676 (U15 male skiers), meaning that 68% of the variability in MEHS can be explained by maturity offset. However, when normalized for body weight, relations between relative MEHS values and maturity offset were not present in U15 female skiers and considerably weakened in U15 male athletes. This suggests that MEHS is strongly dependent on body weight, which naturally increases during puberty.

### 4.4. Methodological Considerations

This study has some limitations that should be considered when interpreting its findings. First, the methodological approach of this study aimed to obtain normative reference values for MEHS in youth competitive alpine skiers in the U10 and U15 age groups. However, the availability of representative skiers and thus the generalizability is certainly limited, although almost all skiers competing in these age groups in Switzerland were tested in this study. Second, NHE-based testing (slow maximal eccentric hamstring strength) may only partially represent the rapid motion threatening the ACL during typical injury mechanisms, even though at least the maximal and eccentric contraction modes seem appropriate. Thus, as suggested by Jordan et al. [34], a plausible complementary assessment approach could be found in rapid, voluntary, and maximal-isometric hamstrings/quadriceps contractions, an approach that would allow maximum torque (MVC) and rate of torque development (RTD) to be determined. Moreover, as it is known that the (pre)activation levels of the hamstring muscles are modifiable and play a crucial role in jump landing-related ACL injury mechanisms [35], the quantification of the activation patterns of the thigh muscles during drop-jump landings as proposed by Ellenberger et al. [36] could also represent a conceivable additional screening approach.

Another limitation is the assessment of hamstring strength in a controlled laboratory environment. Caution is required when transferring the results from the NHE testing to the real-life skiing conditions. The MEHS determined by NHE is a snapshot in time and does not fully reflect the ability of the hamstrings to stabilize the knee, as the quadriceps muscles also play an important role [37]. Finally, it should be noted that according to a previous study, the minimal detectable change of the NordBord test device at a 95% confidence level is 60.1–76.2 N. While the differences between U15 and U10 skiers were larger, differences between the sexes were below this threshold. Thus, the non-significance between the MEHS values of female and male U10 skiers may either be explained by the fact that there is no difference or that the NordBord device has limited sensitivity to existing differences.

## 5. Conclusions

This cross-sectional study established normative reference data for MEHS during NHE in youth competitive alpine skiers and showed that there are sex differences in older skiers but not in younger ones. Based on the present study findings, it is reasonable to conclude that when testing the physical fitness of young athletes around the growth spurt, physical performance depends on the stage of biological maturation, which makes inter-individual comparisons based only on calendar age not really meaningful. In practice, however, athletes of the same chronological age category compete at the same competition level, regardless of their level of maturity. It is therefore important not only to take biological maturation into account but to explicitly focus on it when evaluating and interpreting strength measurements in young athletes shortly before and after the growth spurt. An effective approach in this direction could be to normalize MEHS values with body weight. Moreover, given the apparent significant sex differences in strength at the U15 level, female and male skiers may need individually tailored training regimens to provide adequate training stimuli when training together.

## Figures and Tables

**Table 1:** Baseline and biological maturation data for skiers under 10 years old (U10) and those under 15 years old (U15) separated by sex.

Variables	U10 Skiers			U15 Skiers		
	Overall (*n* = 120)	Female (*n* = 59)	Male (*n* = 61)	Overall (*n* = 126)	Female (*n* = 62)	Male (*n* = 64)
Age (y)	9.4 ± 0.9 (6.9–10.7)	9.2 ± 1.0 (6.9–10.7)	9.5 ± 0.9(7.2–10.7)	13.4 ± 1.4 (10.8–15.7)	13.4 ± 1.3 (10.9–15.7)	13.4 ± 1.5 (10.8–15.7)
Body height (cm)	135.1 ± 7.4 (118.0–157.0)	133.9 ± 7.0 (118.0–157.0)	136.3 ± 7.7 (118.0–152.0)	158.3 ± 10.3 (136.0–187.0)	156.7 ± 8.8 (136.0–177.0)	159.8 ± 11.4 (140.0–187.0)
Body weight (kg)	29.7 ± 4.9 (20.0–50.0)	29.4 ± 5.3 (20.0–50.0)	30.1 ± 4.6 (21.0–41.0)	48.5 ± 10.0 (28.0–90.0)	47.9 ± 9.5 (28.0–65.0)	49.1 ± 10.5 (31.0–90.0)
BMI (kg/m^2^)	16.2 ± 1.7 (13.2–21.8)	16.3 ± 1.9 (13.2–21.4)	16.1 ± 1.4 (13.6–21.8)	19.2 ± 2.2 (15.1–28.4)	19.3 ± 2.2 (15.1–24.7)	19.1 ± 2.3 (15.2–28.4)
Maturity offset (y)	-	-	-	0.2 ± 1.5 (−2.7–3.3)	1.0 ± 1.2 (−1.5–3.3)	−0.6 ± 1.3 (−2.7–2.5)
APHV (y)	-	-	-	13.2 ± 1.0 (11.5–15.7)	12.3 ± 0.4 (11.5–13.3)	13.9 ± 0.6 (12.5–15.7)

Data are expressed as the mean ± SD (min-max). BMI = Body Mass Index, APHV = Age at Peak Height Velocity.

## Data Availability

Restrictions apply to the availability of these data. Data were obtained from Swiss-Ski and are available from the authors with the permission of Swiss-Ski.
